# *“I Think Friendship Over This Lockdown Like Saved My Life*”—Student Experiences of Maintaining Friendships During COVID-19 Lockdown: An Interpretative Phenomenological Study

**DOI:** 10.3389/fpsyg.2022.861192

**Published:** 2022-04-29

**Authors:** Amy Maloy, Annischa Main, Claire Murphy, Lauren Coleman, Robson Dodd, Jessica Lynch, Donna Larkin, Paul Flowers

**Affiliations:** Humanities and Social Sciences Department, School of Psychological Sciences and Health, University of Strathclyde, Glasgow, United Kingdom

**Keywords:** friendship, COVID19, support, lockdown, reciprocity, growth, interpretative phenomenological analysis

## Abstract

COVID-19 lockdown presented a novel opportunity to study the experiences of people attempting to maintain friendships in the context of worldwide, government-enforced physical distancing and lockdown. Here we report on an experiential, idiographic qualitative project with a purposive sample of Scottish students. Data was collected *via* one-to-one on-line interviews with nine student participants (*N* = 9). Data was transcribed and analyzed using Interpretative Phenomenological Analysis (IPA). Analysis highlighted three group-level experiential themes (GETs) and associated subthemes. Participants’ shared experiences of maintaining friendships were reflected in a dynamic process by which (1) ‘changes to communication’ were associated with experiences of (2) ‘effort and balance’ across friendships. Participants reported becoming particularly aware of the psychological processes involved in maintaining friendships, in turn, this was associated with (3) ‘reflection and growth.’ These experiential findings resonate well with several longstanding classic theories; however, they also speak to the particularities of the context in which the study was conducted. They suggest the need for a pandemic psychology that moves beyond the typical focus on the direct impacts of infectious disease to address the wider psychosocial impacts with equal vigor.

## Introduction

Coronavirus disease (COVID-19) was declared a global pandemic on the 11th of March 2020, (World Health Organisation, 2020). Wide-ranging restrictions and various mitigations followed changing the fabric of social lives globally. On the 23rd of March 2020 the United Kingdom Prime Minister announced the country would be entering a nationwide lockdown (Johnson, 2020). People were encouraged to stay at home and avoid mixing physically with others. These unprecedented, profound changes had, and continue to have, deep impacts on people’s lives. This project explores how these changes were felt amongst a small group of students at a Scottish university and how they managed their friendships throughout this period.

Scotland’s first confirmed positive case was the 1st of March 2020, and the first confirmed death was the 13th of March. Government advice began by insisting on self-isolation when experiencing symptoms. Subsequently household lockdowns began on the 24th of March with specific restrictions demanding people ‘stay at home.’ Individuals were asked only to leave their homes to buy food or use pharmacies, spend a maximum of 1 h per day outside for exercise, and to work from home. All schools, nurseries, pubs, restaurants, gyms, and other social venues were closed. Profound changes to daily living were experienced by most (John et al., 2020). Restrictions began to ease on 11th of May 2020 when people were permitted to go outside for more than just essentials. However, people were still required to ‘stay local’ and either go outside alone, or with members of their household only (Scottish Government, 2021). From the 14th of September 2020, the first mixed household gatherings outdoors were allowed although limited to six people from two households. As a result, people had to choose who to spend time with. An increase of COVID-19 cases resulted in the ban of mixed households from the 23rd of September 2020. The constant change in restrictions left many people feeling deflated and finding the ongoing lack of physical contact difficult to manage (Folk et al., 2020). During this time period, online communication became the norm for many social interactions, education, and work. Opinions diverge regarding the quality of online communication. Early work highlighted contrasting ways of thinking about on-line communication. On the one hand, it could compromise the quality of communication (e.g., the displacement hypothesis) yet on the other hand, it could enhance perceptions of intimacy (e.g., the stimulation hypothesis; Kraut et al., 1988; Valkenburg and Peter, 2011). Others noted wider problems with on-line information sharing due to the lack of emotional tone, social presence, and social cues (Daft and Lengel, 1986), these issues remain pertinent today (e.g., Davis et al., 2019). Across this time, mental health trends throughout the United Kingdom tended to worsen often along lines of long standing health inequalities and especially for young adults (Banks and Xu, 2020; Gagné et al., 2021). Studies from the first years of the COVID-19 pandemic show these negative trends related to increasing distress (Farris et al., 2021; Gagné et al., 2021) and loneliness (Branquinho et al., 2020; Singh et al., 2020; Smith and Lim, 2020).

Decades of friendship research shows human beings need a sense of social belonging and emotional support from others (Baumeister and Leary, 1995; Juvonen et al., 2021). Friendships are voluntary and require reciprocal effort to be sustained or they can deteriorate (Wiseman, 1986; Roberts and Dunbar, 2011; Hojjat and Moyer, 2016). Friendships require more effort than family relationships and the emotional intensity of friendships decreases without physical presence (Stadtfeld et al., 2018). A series of large international studies show the positive benefits of close friendship, regardless of age and sex, such as increased social support, life satisfaction, physical health and wellbeing (Amati et al., 2018; Pillemer and Rothbard, 2018; Blieszner et al., 2019; Oh et al., 2020). Friendship amongst the student population can be defined as a dyadic, co-constructed phenomenon characterized by reciprocity, closeness and intimacy (Schneider, 2000; Bagwell and Schmidt, 2011). Student friendship provides invaluable support and is essential to health and wellbeing (Baumeister and Leary, 1995; De Vogli et al., 2007; Holt-Lunstad and Clark, 2014; Snyder et al., 2020; Slavich, 2020), provides happiness while preventing loneliness (Demir et al., 2011; Binder et al., 2012) and during the COVID-19 pandemic seemed to be related to resilience against many adverse impacts (Ang et al., 2021; McKinlay et al., 2022; Owens and Cassarino, 2022). Contrary to these protective factors, recent reviews investigating harm caused by the COVID-19 pandemic on the higher education sector, show that students, overall, experienced increased isolation, greater financial hardship, elevated stress and anxiety due to changes in learning experiences and difficulties in planning for the future (Goodwin et al., 2021; Unterhalter et al., 2021). This may suggest the role of friendship as a protective factor against COVID-19-related harm was socially patterned, affecting some students more than others, or that additional psychosocial mechanisms were at play (Branquinho et al., 2020; Butler-Warke et al., 2021; McKinlay et al., 2022).

A plethora of qualitative research presenting young peoples’ experience during the COVID-19 pandemic details a broad range of problems. These accounts tend to highlight the damaging and potentially long-lasting negative impacts on students, children and young people. These include increases in mental health symptoms and concerns, as well as damage to psychological well being (Branquinho et al., 2020; Evans et al., 2020; Butler-Warke et al., 2021; Farris et al., 2021; Gupta and Agrawal, 2021; O’Sullivan et al., 2021). For those in education, the COVID-19 pandemic brought many barriers to learning (Butler-Warke et al., 2021; Farris et al., 2021; Gupta and Agrawal, 2021). Some studies detail how limited connection (between both staff and students) in the online learning environment adversely impacted individual’s experience of education (Butler-Warke et al., 2021; Lippke et al., 2021; Petchamé et al., 2021), but did not always limit learning *per se* (González-Ceballos et al., 2021). Despite this, some participants overcame this limited connection while in online learning (Ang et al., 2021; Lippke et al., 2021; Owens and Cassarino, 2022).

Other studies chart the negative impacts of social isolation and loneliness on young people and especially students (Branquinho et al., 2020; Butler-Warke et al., 2021; Das et al., 2021; O’Sullivan et al., 2021; Vaterlaus et al., 2021). However, on-going communication that combated the negative social aspects of living through the COVID-19 pandemic was also identified as beneficial and some studies charted a sense of corresponding intra- and inter-personal growth (Branquinho et al., 2020; Evans et al., 2020; Das et al., 2021; Durosini et al., 2021; Vaterlaus et al., 2021; McKinlay et al., 2022; Owens and Cassarino, 2022). Furthermore, some studies report how time recovered from cancelled travel, or other ‘prohibited’ activities, was sometimes used for physical activities that in turn brought a range of benefits for individuals*—*physically, mentally and socially (Branquinho et al., 2020; Lippke et al., 2021).

This current study explored the individual experience of maintaining friendships during the COVID-19 pandemic from the perspective of a group of students. Adding to the growing collection of studies exploring student and young peoples’ experiences during the pandemic, this research attempts to understand how young people maintained friendship during lockdown. Using an in-depth, idiographic qualitative methodology, two men and seven women were interviewed and discussed their experience of maintaining friendships during this stressful period. Here we report on our analysis of this data using interpretative phenomenological analysis (IPA; Smith et al., 2021) and show in great depth and detail what maintaining friendship was like during this period.

## Materials and Methods

### Participants

An opportunistic convenience sample of nine students was recruited *via* social media and personal contact at a Scottish University (academic year 2020–2021). Participants were included in this sample because they responded to the study advert and made themselves available to take part in an online interview. There was no systematic monitoring of uptake rates, but uptake was high, and no participants were excluded. We concluded data gathering after the nine participants due to time restrictions and because a suitable sample size for this type of study had been reached (Smith et al., 2021). Inclusion criteria ensured that participants were over 18 years of age and postgraduate students at the particular university at the time of data collection. There were no further exclusion criteria. Participant demographic information is provided in Table 1.

**Table 1:** Participant demographic information.

Participant pseudonyms	Age (*M* = 26.11)	Gender
Amy	23	Female
Eve	23	Female
Grace	25	Female
Kerry	28	Female
Kiran	36	Female
Patrick	23	Male
Peter	22	Male
Shannon	22	Female
Summer	33	Female

Ethnicity not disclosed to protect anonymity.

### Design

This exploratory qualitative study used the interpretive phenomenological analysis (IPA) approach (Smith et al., 2021) to understand students’ experience of maintaining friendships during the COVID-19 pandemic. Phenomenology is broadly concerned with understanding experience. IPA represents a particular kind of phenomenological approach developed within qualitative psychology that seeks to understand experience through an explicit double hermeneutic process. Firstly, through data collection where typically within interviews, the participants’ make sense of their experience, and secondly, through data analysis as researchers make sense of the participants’ sense-making. Furthermore, IPA can be characterized as distinct from other types of qualitative psychology in that it is concerned with the idiographic (i.e., it has a particular commitment to the individual case) and the experiential (i.e., it is overtly concerned with understanding and interpreting participants’ experiences). It is a particularly useful approach to use at the beginning of a longer process of psychological inquiry especially in a novel area (e.g., student experiences of friendship within pandemic restrictions).

Accordingly, semi-structured, in-depth interviews were conducted (40–70 min), with a relatively homogeneous sample of students. A topic guide (Appendix A) was prepared that focused on the meaning of friendship before and after lockdowns. However, an open-ended, participant-led, approach was taken to data collection that elicited in-depth, first-person accounts of specific thoughts, feelings and experiences concerning friendship within this period. Interviews were conducted by a range of trained interviewers (i.e., the majority of authors). Social distancing restrictions required the interviews to be recorded over Zoom (Zoom Video Communications Inc., 2016). The digital modality of data collection did not impact greatly on the operationalization of typical IPA interviewing techniques (i.e., participant-led interviews being operationalized through active listening, reflecting back, probing). However, it should be noted that the full range of non-verbal body language often used to establish rapport and to communicate experience was somewhat reduced due to the constraints of video conferencing software.

Participants were given the choice to take part with audio only or with the addition of their camera turned on. Interviews were transcribed verbatim. During transcription, participants were given pseudonyms and any identifiable information was omitted/altered to ensure anonymity.

### Analysis

Data analysis followed typical IPA research. However, here we use new terminology (see, Smith et al., 2021).

Each transcript was analyzed independently. It was read and reread to secure a deep familiarity with the data. Secondly, each transcript was explored in greater detail beginning with line-by-line analysis in which exploratory notes were generated considering descriptive, linguistic, and interrogative aspects of the data and notes about participants’ meaning-making. Following this micro-analytic stage, experiential statements were formed from these initial exploratory notes. These experiential statements were intended to capture the participants experience within a more defined, yet still clearly participant-led statement. From here, within each transcript, experiential statements were clustered to form personal experiential themes (PETs) and subsequent subthemes. In this way, for every participant, a detailed understanding of their experiences of maintaining friendship during lockdown was generated. Next, the PETs and sub themes pertaining to each participant, were compared, and contrasted to look for similarities and differences. In turn, these cross-participant analytic entities were clustered to give group experiential themes (GETs), and associated sub themes across the whole sample. Whilst within each GET there was demonstrable commonality between different participants in their experience and meaning making, there were also disparities and contradictions. In the following write up of our analysis, we attempt to highlight this patterning of the group findings.

Initial analysis was conducted individually (each author independently developing PETs and GETs). Subsequently however analysis was collective (iterative weekly discussion and the visualization of findings over 4 weeks). The write up of the analysis was completed by a smaller group of researchers; however, the final analytic structure was agreed by all authors.

Ethical approval was obtained by the School of Humanities and Social Sciences Ethics Committee, University of Strathclyde 41/07/01/2021/A.

## Results

**Figure 1:**
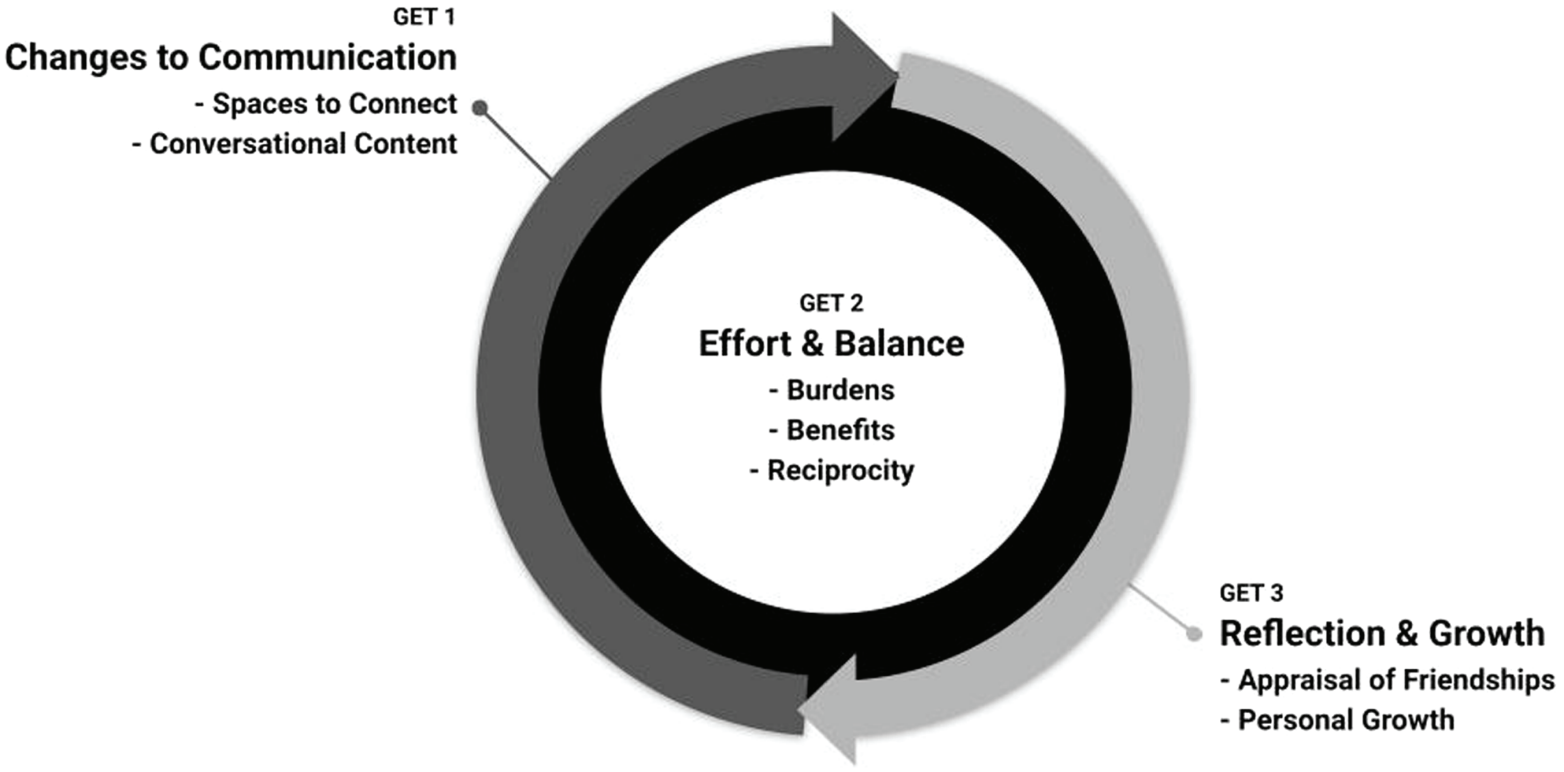
Graphic simplifying the interaction and inter-relatedness of the three group experiential themes (GETs).

The analysis consists of three interrelated GETs. Figure 1 depicts the way the three GETs relate to each other. It shows how contextual factors constrained the modalities of communication (GET1). In turn participants were forced to make sense of friendship in new ways, aware of assessing issues such as effort and balance (GET2). Finally, it shows how these experiences led to a process of reflection and growth (GET3).

### GET 1: Changes to Communication

This GET describes COVID-related changes both in relation to where interactions took place and how participants interacted. All participants talked about the nature and quality of interaction with friends changing profoundly across the lockdown periods with data clustering around two sub-themes ‘Spaces to Connect with friends’ and ‘Conversational content.’

*Spaces to Connect: “Out of sight, out of mind”* (Amy).

The modality of interactions between friends changed dramatically because of lockdown restrictions; from typical in-person to online interactions. The spaces in which people interacted changed dramatically too; from the rich variety of traditional venues such as homes, workplaces and recreational spaces to, initially, a highly constrained repertoire of digital platforms (e.g., Zoom, Teams). Participants’ recounted a strong sense of nostalgia for physical interactions between friends. Peter describes this shared experience:

“You just know it’s not normal, an I think, I think that’s the thing it’s just we are built in a way that we are meant to kinda be close in community with people we are we are meant to be like surrounded by people and like even, even, physically touching people and stuff like that and not kinda worrying about this disease we might pass on but like loving each other and stuff like that so it’s actually, so I think that might be why it like it feels really weird becau…, cos we cannot almost have that, that pure sense of community with our friends or stuff like that.”

Here, Peter outlines a shared inherent need for physical interaction and highlights what was lost within the COVID-19 lockdowns. Peter believes the essence of who we are as people and the benefits of community are at stake. The combination of Pete’s emotive language, “loving” and “pure sense of community,” with the apparent simultaneous dismissal of these ideas, by repeating ‘stuff like that’ perhaps highlights his exasperation and feeling of loss. For many it felt that online interaction was fundamentally removed from real life, Grace explains: -.

“Ehm and then maintaining friends, you are just missing out on all the things that you have done before, but like everyone’s moving on in their life and these are big milestones, and you are just not getting to be involved in them. So, ehm…it’s just been a bit of like… you are there because you see the pictures, but you are just not like physically there, it’s just not the same obviously.”

For Grace the lack of physical connection has highlighted a vicarious quality of being witness to, but seemingly not being part of big events in friends’ lives. Almost as if she is a spirit watching the living from beyond the grave, or as if other people’s lives are on stage and she is the audience always one step removed from ‘real’ life. In contrast, a few participants found online communication, over time, was beneficial, referencing convenience and accessibility.

*Conversational content: “I feel like putting that in a message, […] does not feel the same”* (Eve).

As the previous sub-theme suggested, most participants experienced online interactions in ways that seemed inferior to typical physical interaction. This subtheme explores these issues in more depth. Almost all participants reported interactions getting more superficial and that quality of conversation with friends deteriorated throughout lockdowns. Grace sums this up, reporting that friendships “do not feel as real and authentic as they did before.” Similarly, in the extract below, Patrick begins to outline potential psychological processes that contribute to diminishing authenticity of interactions: -.

“So, it’s like, everyone just seems to be at a place where they are just a bit beaten down. And then there’s the whole trying not to offload too much on each other, because everyone else is feeling the same. But it just kind of leads to these very superficial conversations, so we just kind of suffer on our own and then briefly chat to each other about random stuff, but not anything deeper. So yeah.”

Here Patrick references his close friendship group; him and two others and this extract stresses their mutual awareness of the negative impacts in the on-going COVID pandemic. The usual sources of reciprocal support facilitated through typical interaction have somehow been cut off by the loss felt by his whole friendship group. Furthermore, Patrick’s word choices highlight the slow tortuous experience of lockdown for this friendship group. Contrary to this typical experience of on-line communication, a minority of participants did report the development of meaningful friendships through on-line communication; adjustment over time was key to this.

### GET 2: Effort and Balance

This GET and its subthemes, ‘Burdens,’ ‘Benefits’ and ‘Reciprocity’ shows participants’ awareness of, not only, the benefits of maintaining friendships, but also the concomitant burdens. Overall, it details the delicate balance of the give-and-take that allows friendships to continue and function well. In this way this GET outlines important experiential features of friendship laid bare by the imposed changes to communication.

*Burdens: “..I feel like my oxygen is finished”* (Kiran).

Almost all participants discussed apparent costs in friendship both pre and post lockdown. Kiran said: -.

“And we, we have fun, we have everything with diplomatic way. That’s not more than that. So you can have fun, spend just 1 h, 2 h, not more than that. I can…I feel like my oxygen is finished.”

Here, Kiran’s comparison of spending time with these friends is reminiscent of diving underwater and desperate to return for air. Perhaps the “oxygen” she craves is to be herself, without managing herself within her interactions. The extract suggests such interactions have an element of performance, there is a publicly visible Kiran being managed by the real and exhausted Kiran somewhere else. Below, Grace outlines a different perspective and shows how she pressures herself to be fully present within her on-line interactions:-.

“.. I think that you feel a sort of pressure to maintain them. Ehm, then you feel sort of guilty, if you are having like an off day, […]..because it’s this pandemic and because it’s so hard, like, on other people, with everyone you feel like, ‘Oh, I need to make sure that I’m like really present and there for people.”

Most participants reported being drained by the lockdowns and at times feeling obliged to engage in friendships. Friendship burden related to issues such as energy expended, fulfilling a sense of duty, or managing sometimes self-imposed expectations about authenticity.

*Benefits: “They would be there for me”* (Patrick).

Alongside burdens, all participants also recognized the benefits of friendship. Kiran, for example, stresses their centrality to her maintaining her mental health:

“because I think friendship over this lockdown like saved my life. Because you need people just to talk with them and like to share with you your future plan […] So they became more important than my past life.”

When the present was difficult having people to talk to about the future was particularly important. Similarly, Summer also stresses the profound positive value of friendship. She draws on the notion of self-sacrifice to illustrate the importance of friendship within the lowest points, the dark days, of living through COVID-19 restrictions saying:

“Yeah, like I say, I’m quite lucky, the friends that I’ve got, you know, that old saying, when people say, you know, ‘they would lie in front of traffic for you,’ I’ve got those types of great friends who would do that for you and I would do it for them. […] There’s been times where, you know, there’s been dark days through lockdown […] And I have loads of, you know, people checking in, me checking in on them, being able to talk probably having maybe more deep conversations than we might have had otherwise.”

*Reciprocity: “They’re always there. And I’m always there for them.”* (Patrick).

The third subtheme highlights that burdens and benefits should balance. For most participants there was a strong sense that reciprocity was fundamental to friendship maintenance. Below Patrick talks of his closest friends:

“And they were the ones that were there consistently throughout. […]. So, I think yes, it’s kind of like a, dare I say, ‘safety net.’ And I feel like a ‘safety nets’ a very degrading, way of referring to them. But as it that’s what it feels like, it’s that they are always there. And I’m always there for them. So yeah….”

Patrick’s use of the word “there” implies a transcendental safe place where he and his friends are available to provide unconditional and immediate support to each other.

### GET 3: Reflection and Growth

This GET focuses particularly on the psychological processes concerned with the appraisal of friendship. COVID-19 rendered these processes visible in new ways. We identified two sub-themes: - ‘Appraisal of Friendships’ and ‘Personal Growth.’

*Appraisal of Friendships: “it just felt like there was no effort being put in”* (Amy).

All participants reported that some friendships dissipated over lockdown; how and why this occurred differed. The key commonality we address within this subtheme concerns how participants became much more aware of their active appraisal and monitoring of friendships during COVID restrictions. As Amy explains:

“I really noticed who my friends were and who I probably would not speak to after lockdown was over who I would not go back and socialise with because it yeah.”

Amy alludes to how the context of COVID–19 seemed to illuminate key psychosocial processes associated with friendship in ways distinct from pre-COVID times. This heightened sense of awareness concerning the appraisal of friendships peppered many of the participants’ accounts. As Summer says:

“…. loyalty is probably the one that I think, it’s just a funny word, is not it?.. Because I do not think my friends who have not hung out with or being disloyal to me by selecting other people. But I guess you could be like, ‘Well, why not me?’ ‘Why did you not ask me if I wanted to come?’ But then I suppose for me, that takes me down quite an unhelpful thinking path..of comparing.”

The internal monologue within Summer’s account highlights her awareness of her active appraisal of her friendships. Her assessment centres on questioning her relative value in her impoverished social world. However, the downplaying of negative emotions, e.g., calling it “funny,” allows a positive reflection that this is an “unhelpful” path. Pete, below, shows how COVID-19 restrictions have made him revisit his own expectations and sense of duties as a friend:

“. I think these times obviously highlighted this, the whole idea of actually … reaching out more. I should be talking to this person more … If I actually view them as a friend, then, ….maybe I should show up a bit more and reach out a bit more. Cause it’s funny that you call … people friends that you might not even like speak to …in a year, ….like some people you cannot speak to maybe like so often. But it is funny, that you would like maybe see them at like, … church events and stuff like that, and like I’d call maybe all of them my friends but …am I, actually …showing it? (laugh) ….I guess it’s changed that meaning, in terms of actually like ‘if you are gonna call ‘em your friend then actually you, kinda reach out and show up even when you do not have to.”

Pete’s ideas crystalize around talking with friends, being with friends if possible, and ‘reaching out’ to them proactively (this phrase centres on being prepared to save someone by literally pulling them out of a difficult situation). Others, such as Amy, became aware of how friendships could be appraised, and expectations could be nuanced. “She did want to see me, but her parents are more important. Which is like perfectly understandable I would not hold that against her….”

*Personal Growth: “...forced me to realise whats important in a friendship”* (Grace).

This sub-theme highlighted ways in which participants learned from their friendship experiences and how these changes may affect their future perspectives. All participants recognized the importance of friendship, gaining a greater appreciation for them, “because it kinda weeds out the unimportant stuff” (Shannon). The context of COVID-19 restrictions seemed to distil the value of friendships and enable participants to learn and grow:

“So let us say that we lived in the normal world, no pandemic and these people suddenly started drifting away, that would have really bothered me.”

Participants emphasized the importance of their realization that support, especially in times of crisis, could help to alleviate anxiety and improve mental health. Grace summed this up saying:

“so, I think it’s just really, the importance of the friendship and the support system has just been highlighted throughout the pandemic. Like my friends have been the thing I have held so closely and have been so important in like making me feel happy and loved and supported. So like yeah… as much as they have been really difficult to maintain sometimes, I think the effort I have put into them to maintain them has just like confirmed how important they are to me.”

Grace’s extract emphasizes her newfound awareness of the importance of friendship support, how love and support had helped her cope with lockdown conditions.

## Discussion

Our analysis highlighted three interdependent group experiential themes (GETs) that reflect the experience of maintaining friendships through COVID-19 restrictions. Each GET focused on a spectrum of shared experiences across the nine participants. We obtained a rich, detailed and experiential understanding of maintaining friendships during this time. The first GET related to how changes in communication were experienced, the second focused on key mechanisms of friendship maintenance and the final GET illustrated psychological processes concerned with friendship evaluation and personal growth. Our analysis offers a valuable contribution to both the friendship and the emerging COVID-19 literature; some findings resonate with longstanding theories, while others provide novel perspectives regarding friendship maintenance in the context of profound social change and high levels of population level distress. Most significantly, IPA facilitated the exploration of experiences and meaning in depth and detail, offering a uniquely idiographic perspective on maintaining friendships during the COVID-19 pandemic.

Our first GET highlighted the importance of the enforced and changing modalities of communication between friends. This was a prominent feature of the COVID-19 restrictions and unprecedented levels of enforced population-level digital communication were taking place across the world. For many, and for the first time in human history, this became the primary modality through which people could interact. Our participants spoke of concomitant changes to interaction content and sense of connection. Across the participants, these changes were experienced as both negative and as positive, and as changing over time. Echoing earlier pre-COVID findings our participants stressed that on-line communication when compared to face-to-face communication was sometimes experienced as inauthentic and shallow (Kraut et al., 1988) and somehow lacking in value and depth (Lippke et al., 2021). This is perhaps unsurprising as lockdown moderated what can be thought of as fundamental friendship maintenance behaviors. For example, social distancing limited friends’ ability to participate in many joint activities putting the friendship at risk of decay (Lippke et al., 2021). This relates to findings from wider friendship research and theory (Roberts and Dubar, 2011). Conversely, in relation to the positive aspects of these changes to communication, some participants highlighted an emerging sense of greater closeness, intimacy and meaning matching the findings of other online friendship research (Valkenburg and Peter, 2011). In the context of the pandemic, this may indicate that having access to online communication during isolation allowed people to find growth within friendships, echoing McKinlay et al. (2022) findings. We found this tended to occur over time. Our participants’ divergent experiences can be illuminated by population-level heterogeneity in Online Communication Attitudes (OCAs; Ledbetter, 2009; Ledbetter et al., 2011) although it should be noted that the context of restrictions precluded other means of communication for many people. Similarly, historically gender has shaped differences in online-disclosure which moderates feelings of emotional closeness and social connection (Valkenberg and Peter, 2011; Muscanell and Guadagno, 2012; Kimbrough et al., 2013; Casale and Fioravanti 2015). Further, our participants alluded to differing experiences of the online learning experience. Here they focused on the lack of social experiences available, rather than the learning experience itself. This is interesting as other research has found that online learning is not always academically a disadvantage (González-Ceballos et al., 2021) even though it was commonly viewed as a hindrance to the learning experience (Butler-Warke et al., 2021; Petchamé et al., 2021).

Our second GET relates to effort and balance within friendships. Reciprocity and supportiveness were important, a finding reflected within the pre-COVID literature (Trivers, 1971; Hall, 2010; Demir et al., 2012; Blieszner, 2015; Chopik, 2017). Mutual expectations of support and reciprocity have long been understood as fundamental aspects of friendships (Blieszner., 2015; Hall., 2010; Amati et al., 2018). These aspects of friendships are reflected within classic theories such as Social Exchange Theory, which posits a cost–benefit framework for relationships whereby individuals will only engage in relationship maintenance behaviors if the perceived rewards outweigh the expenditure (Emerson, 1976; Huston and Burgess, 1979). This was true for many of our participants; the onerous effort of friendships was overridden by the support and encouragement that they offered. Previous lockdown research has highlighted that the support of friendships bolstered resilience during the pandemic (Ang et al., 2021; McKinlay et al., 2022; Owens and Cassarino, 2022), which is in line with our participants’ perceived willingness to expend effort, and gratitude for what their friends returned to them. Although our participants did appear to value friendships and notice benefits from them, they often expressed that they were facing challenges in lockdown despite having friendships which is congruent with research suggesting that friendship was not enough to mitigate negative impacts of lockdown and the pandemic (Branquinho et al., 2020; Butler-Warke et al., 2021; McKinlay et al., 2022). Reciprocity was understood to be implicitly embedded within friendship maintenance. Defined as a major moral feature of friendships (Pahl, 2000), it is suggested that “individuals are motivated to perform relational maintenance behaviors as long as they see their relational investments reciprocated” (p.175, Rousseau et al., 2019). Indeed, participants described a willingness to expend their increasingly finite resources of support and maintenance only when they were reciprocated, creating a relationship fueled on cyclical give-and-take. Across time, some friendships were also experienced as becoming increasingly less connected and more distant, with breakdown of reciprocal effort often reported. While reciprocity was intertwined with burdensome responsibility for some, others spoke forgivingly of friends’ lapse in reciprocating effort during the pandemic, suggesting a longer-term vision of reciprocity whereby only small gestures were enough to maintain friendship.

Our final GET was concerned with appraisal and growth. The COVID-19 context drove a heightened awareness of the psychological processes associated with maintaining friendships: in the impoverished communication context of the online world participants became particularly attuned to the balance of perceived effort and balance of friendship. The growing salience of these processes could relate to the fact that overtime people became increasingly fatigued with COVID-restrictions and the enforced communication changes. The latter focused a spotlight on the underlying mechanisms of friendship compelling individuals to question the costs and benefits of their relationships. This resulted in an attenuation of many friendships, creating a process of ‘thinning the herd,’ whereby individuals were forced to consider the value of each of their friends (echoing key tenets of Social Exchange, Huston and Burgess, 1979). It remains unclear what the long-term repercussions of this relational vigilance will be. Moreover, the appraisal of friendships often led participants to experience personal growth, whereby new insights into themselves were gleaned and a greater appreciation for certain friendships occurred. This resonates with the findings of multiple other lockdown studies (Branquinho et al., 2020; Evans et al., 2020; Das et al., 2021; Durosini et al., 2021; Vaterlaus et al., 2021; McKinlay et al., 2022; Owens and Cassarino, 2022) suggesting that both personal growth and growth within relationships were described when communication was upheld, furthermore, as well as echoing others that suggest that the COVID-19 pandemic and enforced isolation could be traumatic but lead to post-traumatic growth (Evan, et al., 2020; Tamiolaki and Kalaitzaki, 2020). The personal growth expressed by participants is in line with similar research during COVID-19 (Evans et al., 2020; González-Ceballos et al., 2021), which suggests that many meaningful learning experiences were facilitated by social interactions despite these being distanced.

Our study shows how the context of COVID-19 highlighted a collective feeling of friendship resources becoming noticeably finite, in low supply and high demand. This chimes with quantitative findings, for example Naser et al. (2020). The expenditure of friendship resources was dependent upon the perceived quality and strength of friendships, with participants more willing to commit effort and support to highly valued friendships. These findings echo earlier literature (Pahl and Spencer, 2004; Rözer et al., 2015). Individuals tend to engage in greater maintenance behaviors with emotionally close friends than casual acquaintances both prior to (Roberts and Dunbar, 2011; Binder et al., 2012) and during COVID-19 (Branquinho et al., 2020; Elmer et al., 2020). In part our findings also speak to the idea of post-traumatic growth (Tedeschi and Calhoun, 1996; Joseph, 2012). Some participants experienced personal growth, or grew in some way with friends, during these difficult times (Tamiolaki and Kalaitzaki, 2020; Bridgland et al., 2021). Our findings also provide qualitative insight into growth in relationships (Stallard et al., 2021). Our analysis, charting how participants made sense of managing friendship across lockdown, adds a unique perspective to recent work that shows the protective nature of friendship during the COVID-19 pandemic (Slavich, 2020; Ayers et al., 2021). The passing of time appeared to play a significant role in experiences of connecting online; for some, time fostered greater intimacy, while for others, time eroded conversational depth. The significance of time is reflected in recent literature; Folk et al. (2020) reported that individuals’ sense of social connection remained largely intact following just one month of lockdown measures, however after three months, Lippke et al. (2021) reported that individuals were not fulfilled with their online social interactions, identifying a need for ‘real connection.’ This is in-keeping with research finding that social isolation and loneliness were increased during the pandemic (Branquinho et al., 2020; Butler-Warke et al., 2021; Das et al., 2021; O’Sullivan et al., 2021; Vaterlaus et al., 2021) despite access to online communication. For many participants, the absence of physical presence was associated with feelings of loss and disconnect, suggesting time and grief-processes may be pertinent concepts in understanding friendship maintenance (e.g., Stroebe and Schut, 1999). These insights are reflected by the Australian Psychological Society (2020) who explained that prolonged lockdowns may cause significant fatigue and exhaustion which worsens with time (Meo et al., 2020), leading to loneliness (Singh et al., 2020) and a lack of enjoyment in activities (Margaritis et al., 2020) and motivation (Kapasia et al., 2020). Taken together, these experiences of grief and fatigue may give some insight into the significance of time in participants’ experiences of connecting online with friends.

### Strengths and Limitations

A key strength of the present study is that it used an IPA approach (Smith et al., 2009, 2021). This facilitated the collection of participant-led, in-depth accounts of the lived experience of maintaining friendships during the COVID-19 pandemic. The choice of IPA and its commitment to the idiographic enabled the gradual development of our findings based on in-depth, case by case analysis to move toward our broader understanding of the shared experience of maintaining friendships during COVID-19 restrictions. However, there have been a number of other studies into the effects of the COVID-19 pandemic adopting different qualitative methods of analysis in order to gain a greater understanding of the attitudes and experiences of the student population. These studies often used different forms of Content analysis (Das et al., 2021; González-Ceballos et al., 2021), Thematic analysis (Evans et al., 2020; Ang et al., 2021; Farris et al., 2021; McKinlay et al., 2022; Owens and Cassarino, 2022) or a combination of the two (Gupta and Agrawal, 2021). This methodology tends to focus on themes found within the data, whereas Interpretative Phenomenological Analysis (IPA) framework used in this study, adds to this, exploring patterns of meanings across all participants while also capturing the individual lived experiences. Therefore, it is this dual focus that contributes to the examination of the complex nature of living through a pandemic.

As interviews were conducted 1 year into COVID-19 restrictions participants could consider their experiences of maintaining friendships through a long recall period. The study took place whilst the whole research team were also living and working through the same circumstances as their participants and arguably this brought both strengths and potential weaknesses to the study. In relation to strengths all the team had firsthand insight and their own experience to help interrogate and analyze the participants’ experiences. The use of the analysts’ own experience is actively encouraged within IPA as a means of appraising what the experience was like for each participant. Reflexive thinking and interpretation are central to using IPA well. Furthermore, given the collective nature of this particular analysis, everything was considered and discussed at length within the group. However, the team were essentially working within their own virtual bubble. Weaknesses relate to the limits of confident transferability of our findings beyond the National time and temporal context in which the study took place. Whilst we are confident that our analysis reflects the experiences of our participants and is likely to reflect the experiences of students and young people elsewhere within Scotland at the time of data collection the wider parameters of transferability are unclear. Further research could conduct similar IPA studies in different national contexts and with different age groups. It may be particularly important, for example, to explore how friendship maintenance occurred and can recover amongst an older generation relatively unfamiliar with, or lacking access to, online communication platforms. Alternatively, the impact on friendship maintenance for individuals who did not have a shared interest to relate to their friends with (i.e., class work and university) could also be explored for differences. Furthermore, in the future, mixed methods and longitudinal designs could be adopted to triangulate diverse kinds of findings (e.g., IPA, quantitative, discursive approaches) and offer holistic and long term insights into the phenomena of friendship maintenance. Such a programmatic approach may well enable the generation of further theory.

In relation to recommendations for practice, given the nature of the IPA study (e.g., depth not breadth), our findings can only tentatively suggest ways of assisting with student health and wellbeing. It may well be that institutions, communities, and families all need to develop innovative ways of re-establishing and securing the maintenance of friendships lost through COVID-19 as suggested by Singh et al. (2020). In relation to this, it may be useful to think of ways of co-producing scalable interventions that celebrate and share the insights, personal growth and resilience gained by some, to begin to enable the reduction in inequalities that have been amplified by COVID-19. Alternatively, findings may provide more insight into digital communication and inform interventions or the promotion of social connection online.

## Conclusion

Using experiential, idiographic, qualitative methods this study sheds particular light on the experience of maintaining friendships during COVID-19 lockdowns. It presents a novel and unique contribution to literature. It reported the psychological processes of friendship maintenance within this context as felt by one small sample of Scottish students. The findings chime with many long-standing psychological theories, particularly those relating to personal relationships and to some extent grief and post-traumatic growth. However, the particular context of living through restrictions seemed to have brought many of the fundamentals of friendship to the surface enabling people to experience, question and consider what friendship means and how to maintain it. Whilst our analysis speaks volumes about the experiences of the participants who took part in the study, the transferability of its findings to other populations remains unclear. Moreover, the study begs questions about the need for, and nature of, a broader pandemic psychology that moves beyond a focus on the direct effects of infectious disease but seeks to understand how pandemics mediate typical psychological processes. This latter focus is likely to be particularly useful for understanding the long-term effects of pandemics.

## Data Availability Statement

The raw data supporting the conclusions of this article will be made available by the authors, without undue reservation.

## Ethics Statement

The studies involving human participants were reviewed and approved by School of Humanities and Social Sciences Ethics Committee, University of Strathclyde. The patients/participants provided their written informed consent to participate in this study. Written informed consent was obtained from the individual(s) for the publication of any potentially identifiable images or data included in this article.

## Author Contributions

This Authorship order was generated randomly as all Authors believe we all contributed equally to this project. All authors conducted research and analysis of their own participant and contributed to the content and editing of this study. All Authors met regularly for project inception and group analysis, however, RD, AmM, and CM continued the analysis from these meetings and completed the written up analysis. DL and LC wrote, edited and finalized the introduction. AnM wrote and finalized the methods section, with editing comments from RD. JL, AnM and RD wrote the discussion section, with editing from LC, AmM, and CM. PF advised the authors throughout research and write-up, assisted in group-level analysis and conducted regular editing—including the final edit of the document. All authors edited and signed off on the final version of this study. All authors contributed to the article and approved the submitted version.

## Funding

Funding for publication was provided by the School of Psychological Sciences and Health at the University of Strathclyde.

## Conflict of Interest

The authors declare that the research was conducted in the absence of any commercial or financial relationships that could be construed as a potential conflict of interest.

## Publisher’s Note

All claims expressed in this article are solely those of the authors and do not necessarily represent those of their affiliated organizations, or those of the publisher, the editors and the reviewers. Any product that may be evaluated in this article, or claim that may be made by its manufacturer, is not guaranteed or endorsed by the publisher.

## Appendix

### Appendix A: Topic Guide

**Background to the interview.**

**Start with Brief Overview:**

·I’m X, I’ll be conducting this interview today. How are you? What are your pronouns? Are you comfortable?

·This interview should be no longer than 1 h, when we get near the end if there’s still things to discuss we’ll see if we want to continue. Is that all okay for you? Are you available for that?

·Interviews can be weird- it’s not like therapy or like chatting to a friend but also not a conversation with a professional or job interview. It’ll be a very one-sided conversation where I’ll mainly be asking you to tell me more about things you say or clarifying that I understand correctly what you are describing.

·Everything we say here will be confidential unless it breaches a boundary (e.g., risk of harm). And we are going to be recording today so that we can create a written account of what you say. All of the data will be securely stored and anonymised. Is that okay?

**Consent Form:**

·Check they have signed and sent you the Participant Consent Form.

·Check they have understood it & what this study is about.

·Do you have any questions about the study? Or anything you did not understand in the documents I sent you?

·Can I collect a few Demographic details from you? These are for the research team to ensure we can explain who this study has been with across the different interviews we gather.

**Demographics:**

·Sex? Gender? Age? What year of Uni? Where in the world are you just now? Nationality?

Summary of the Study:

·This study is about gathering your experience and thoughts on maintaining friendships during lockdown. We want to hear how you found it and dealt with it, not what you heard or someone else did*—*but how it was for you.

·Before we start*—*Are you ready? Are you okay with me turning on the recording equipment now?

Can you tell me about what friendship meant to you before lockdown?

Can you tell me how you have maintained your friendships since March 2020?

**Indicative prompts:**

·Tell me about any thoughts you have had about the nature of friendship?

·Tell me about any thoughts you have had about the meaning of friendship?

·Tell me about any thoughts you have had about the significance of friendship?

Can you tell me what it’s been like for you to maintain your friendships since March 2020.

**Indicative prompts:**

·Can you tell me about any ups or downs?

·How about any different ways of connecting?

·How about any challenging times in maintaining friendships?

·What’s made it easy for you to maintain friendships?

·What’s made it hard for you to maintain friendships.

Have you experienced friendship differently during lockdown?

**Indicative prompts:**

·How did the friendships change?

·Can you tell me about any positive effects?

·Can you tell me about any negative effects?

·Can you tell me how your friendships have related to your mental health?

·How did you find the experience of different ways of connecting?

·What was the impact of this interaction with friends on you?

Has the meaning of friendship changed for you at all since lockdown?

·How.

·Details.
